# Comparison of Transperineal Mapping Biopsy Results with Whole-Mount Radical Prostatectomy Pathology in Patients with Localized Prostate Cancer

**DOI:** 10.1155/2014/781438

**Published:** 2014-05-11

**Authors:** Darren J. Katz, Rodrigo Pinochet, Kyle A. Richards, Guilherme Godoy, Kazuma Udo, Lucas Nogueira, Angel M. Cronin, Samson W. Fine, Peter T. Scardino, Jonathon A. Coleman

**Affiliations:** ^1^Urology Service, Department of Surgery, Memorial Sloan-Kettering Cancer Center, 1275 York Avenue, New York, NY 10065, USA; ^2^Urology Department, Western Health, Gordon Street, Footscray, VIC 3011, Australia; ^3^Men's Health Melbourne, Centre for Specialist Men's Health and Fertility, Level M, 233 Collins Street, Melbourne, VIC 3000, Australia; ^4^Department of Urology, Clinica Alemana, Avenida Manquehue Norte 1410, Vitacura, Santiago, Chile; ^5^Cadence Health, Central Dupage Hospital, 25 North Winfield Road, Winfield, IL 60190, USA; ^6^Baylor College of Medicine Medical Center, 7200 Cambridge Street, Suite 10B, Houston, TX 77030, USA; ^7^Department of Pathology, Memorial Sloan-Kettering Cancer Center, 1275 York Avenue, New York, NY 10065, USA; ^8^Department of Urology, Faculty of Medicine, Saga University, Nabeshima 5-1-1, Saga 849-8501, Japan; ^9^Minas Gerais Federal University, Avenida Alfredo Balena 110, 30130-100 Belo Horizonte, MG, Brazil; ^10^Department of Epidemiology and Biostatistics, Memorial Sloan-Kettering Cancer Center, 1275 York Avenue, New York, NY 10065, USA; ^11^Division of Population Sciences, Department of Medical Oncology, Dana-Farber Cancer Institute, 450 Brookline Avenue, Boston, MA 02215, USA

## Abstract

*Objective*. We sought to evaluate the accuracy of transperineal mapping biopsy (TMB) by comparing it to the pathology specimen of patients who underwent radical prostatectomy (RP) for localized prostate cancer.* Methods*. From March 2007 to September 2009, 78 men at a single center underwent TMB; 17 of 78 subsequently underwent RP. TMB cores were grouped into four quadrants and matched to data from RP whole-mount slides. Gleason score, tumor location and volume, cross-sectional area, and maximal diameter were measured; sensitivity and specificity were assessed.* Results*. For the 17 patients who underwent RP, TMB revealed 12 (71%) had biopsy Gleason grades ≥ 3 + 4 and 13 (76%) had bilateral disease. RP specimens showed 14 (82%) had Gleason scores ≥ 3 + 4 and 13 (76%) had bilateral disease. Sensitivity and specificity of TMB for prostate cancer detection were 86% (95% confidence interval [CI] 72%–94%) and 83% (95% CI 62%–95%), respectively. Four quadrants negative for cancer on TMB were positive on prostatectomy, and six positive on TMB were negative on prostatectomy.* Conclusion*. TMB is a highly invasive procedure that can accurately detect and localize prostate cancer. These findings help establish baseline performance characteristics for TMB and its utility for organ-sparing strategies.

## 1. Introduction

Patients who are candidates for organ-sparing management of prostate cancer, including active surveillance or focal therapy, are optimally risk-stratified and counseled when there is accurate knowledge of the extent, location, and true grade of their cancer. While the treatment and management of prostate cancer is often conceptualized on the assumption of its multifocality, studies of radical prostatectomy (RP) specimens have shown that approximately 20%–30% of men with prostate cancer have unilateral or unifocal tumors amenable to focal therapy; furthermore, small contralateral tumors often represent incidental, indolent lesions [1].

Standard transrectal prostate biopsy strategies, including extended and saturation biopsies, have been developed with the intention of improving prostate cancer detection but not to accurately locate or provide biometric data to characterize or stage prostate tumors [2]. Biopsy techniques that more accurately and systematically map prostate cancer foci are less prone to sampling errors and may provide better biometric data for improving outcomes of organ-sparing management strategies. Template-guided transperineal mapping biopsy (TMB) of the prostate has the potential to provide more accurate information regarding tumor grade, spatial distribution, and local extent of disease.

Previous studies using TMB have provided information on biopsy outcomes, theoretical modeling, and ex vivo sampling regarding location and grade [3–5], but the direct association between clinically obtained TMB findings and the matching prostatectomy pathology has not been described. The purpose of this study is to evaluate the accuracy of TMB by comparing it to the final pathology specimen in patients with localized prostate cancer who underwent radical prostatectomy.

## 2. Methods

This was a retrospective analysis utilizing the institutional prospective database at our tertiary care center; institutional review board approval was obtained. Seventy-eight patients underwent TMB from March 2007 to September 2009. Indications for TMB were either a previous diagnosis of low-volume or low-grade prostate cancer prior to a definitive management decision (*n* = 68) or a rising prostate-specific antigen (PSA) and previous negative transrectal ultrasound (TRUS) biopsy (*n* = 10). Of these 78 patients, 17 subsequently underwent RP. Fifteen were initially candidates for active surveillance based on prior TRUS biopsy (cutoff was clinical stage ≤T2a disease with a maximal length of cancer in each core ≤2 mm) but elected for RP based on increased grade (*n* = 7) or volume (*n* = 8) of prostate cancer as reported by TMB. The other two men underwent TMB for clinical suspicion of prostate cancer following prior negative biopsy.

TMB was performed under sedation using a brachytherapy template with 5 mm spacing and an automated biopsy gun with an 18-gauge needle and 19 mm core sampling length (Bard Magnum and MN1825, respectively, Bard Biopsy Systems, Tempe, AZ, USA). A modification of the Barzell technique was used [6]. The prostate was divided into 16 zones, including anterior versus posterior, medial versus lateral, right versus left, and apical versus basal, according to a standardized template. The cores were taken every 5 mm; therefore, the number of biopsy cores obtained in each procedure was dependent on the volume of the prostate gland according to intraoperative ultrasound.

**Figure 1:**
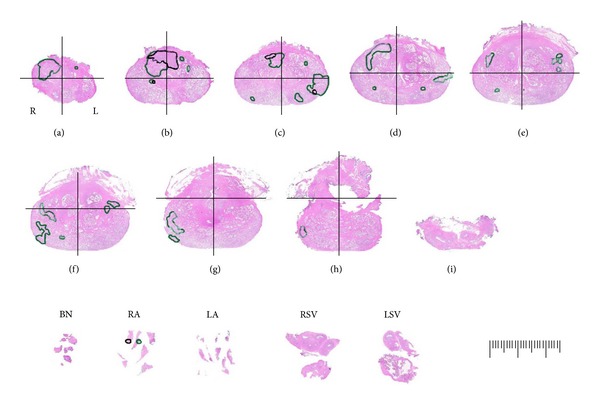
Representative example of study tumor map from whole-mount slides obtained in 3 mm sections. Colored ink outlines tumor location (green = Gleason pattern 3, black = Gleason pattern 4 or 5).

To minimize the risk of localizing errors in the longitudinal plane (*Z*-axis), TMB results were grouped into four quadrants (left anterior, right anterior, left posterior, and right posterior). The anteroposterior plane was defined by a transverse line crossing the urethra, precisely at the level of the anterior tip of the verumontanum in the midapical sections. A pathologist reviewed and provided detailed analysis of digitized tumor maps from RP whole-mount slides obtained in 3 mm sections provided Gleason score, tumor location and volume, cross-sectional area, and maximal diameter (see Figure 1). Whole-mount specimens were processed as previously described, obtaining 3 mm sections perpendicular to the rectal plane from apex to base with separate evaluation of apex and bladder neck margins by radial sectioning [7]. Digital reconstructions were created from photomicrographs as previously published with anterior/posterior plane established using the urethra at the midprostate [8]. Whole-mount data were matched to TMB quadrants, resulting in a total of 68 quadrants for the analysis. A significant tumor in a TMB quadrant was defined as a Gleason score ≥7, >50% involvement of a single core, or >25% of total cores positive for cancer. A significant cancer or index tumor in the prostatectomy specimen was defined as tumor volume >0.5 cc and/or a Gleason score ≥7.

### 2.1. Statistical Analysis

The sensitivity and specificity of the TMB for detection of prostate cancer were calculated using pathologic analysis of the prostatectomy specimen as the gold standard. Estimates were calculated on the specimen level (*n* = 17) and at the quadrant level (*n* = 68), with each quadrant treated as an independent specimen. Since this cohort was small and highly selected, these results should be interpreted in the context of the feasibility of TMB and may not be appropriately generalized to the population of patients with low-risk prostate cancer. Statistical analyses were conducted using Stata 10.1 (StataCorp LP, College Station, TX, USA).

## 3. Results

Table 1 summarizes patient and tumor characteristics at initial TRUS-guided biopsy (*N* = 17). All patients with tumor on TRUS biopsy (*n* = 15) had clinical stage ≤T2a disease and a maximal length of cancer per positive core of ≤2 mm. There were two patients in whom the initial TRUS biopsy did not detect tumor. Three of 17 patients (18%) had biopsy Gleason scores of 7 (3 + 4), and the others had Gleason scores ≤6. Only one patient was reported to have bilateral disease by TRUS biopsy.

Table 2 summarizes tumor characteristics on the basis of TMB and prostatectomy specimens. The median core density from the TMB was 1.26 (interquartile range IQR 1.08–1.51). On the basis of the TMB, 12 patients (71%) had biopsy Gleason scores >6 and 13 (76%) had bilateral disease. On the basis of the prostatectomy specimen, 14 patients (82%) had Gleason scores >6 and 13 (76%) had bilateral disease.

Details of the 17 patients' TMB results and prostatectomy specimen results are summarized by quadrant in Table 3. Approximately half of the patients had 20 or more cores taken from each quadrant. From the 68 quadrants examined, 38 were positive for cancer on TMB and 42 were positive in the final pathology specimen. Overall, the sensitivity and specificity of TMB for detection of prostate cancer were 86% (95% confidence interval CI 72%–94%) and 83% (95% CI 62%–95%), respectively (Table 4). There were no obvious differences in the operating characteristics of the TMB when analyzed separately by quadrant, as the CIs were very wide and overlapping. There were four quadrants in which cancer was present but not detected by TMB. The tumor volume and Gleason scores for the four false negative quadrants were 0.07 cc, 3 + 3; 0.26 cc, 3 + 3; 0.39 cc, 3 + 4; and 0.52 cc, 3 + 4, respectively. There were six false positive results—quadrants where cancer was detected on TMB but not in the final pathologic specimen. Four of six false positive results were Gleason 3 + 3 tumors. However, tumor was detected in the adjacent ipsilateral quadrant in all but one false positive area, suggesting errors in targeting and not errors in pathologic evaluation.

Fourteen of 17 cases in this series revealed index tumors (>0.5 cc and/or Gleason score ≥7) in the final pathology specimen. From the 68 radical prostatectomy quadrants, 42 were positive for cancer, of which 26 were considered significant cancer (>0.5 cc and/or Gleason score ≥7). The sensitivity and specificity of TMB to identify a significant tumor in these prostatectomy quadrants were 92% and 52%, respectively. The positive predictive value and negative predictive value of TMB for significant tumors in this series were 66% and 86%, respectively. When correlating to significant features per TMB quadrant (Gleason score ≥7, >50% involvement of a single core, or >25% of total cores positive for cancer), sensitivity and specificity were little changed, 81% and 74%, respectively.

## 4. Discussion

Tumor multifocality and identification of biologically aggressive cancers are a central issue in the management strategies developed to evaluate and treat prostate cancer.

Appropriate selection of candidates for organ-sparing options, such as active surveillance or focal therapy, requires reasonably accurate knowledge of the extent, location, and grade of the patient's cancer. As yet, no optimal imaging study is reliably capable of this task. Standard TRUS-guided biopsy strategies have evolved from a 6-core approach [9] to more extended strategies, including 12-core and saturation (20-plus core) biopsies, with a cancer detection rate of approximately 40–45% [10, 11]. However, these office-based strategies were developed to improve overall detection of prostate cancer, and they yield suboptimal information about location, Gleason score, and extent of tumor [2, 12]. As a result of the associated uncertainties, many patients with low-risk disease choose treatment and, conversely, some men inappropriately elect surveillance.

Studies in patients with localized prostate cancer diagnosed after an initial standard TRUS-guided biopsy and treated with radical prostatectomy have found that biopsy Gleason score is upgraded 30%–50% of the time based on evaluation of the entire prostatectomy specimen [13]. Epstein et al. [14] evaluated the prostatectomy specimens of 103 men believed to have insignificant cancer based on an initial TRUS-guided biopsy. They found that 29% of these men had higher-volume and/or higher-grade disease than expected. The identification of only a single, or unilateral, focus on traditional sextant or extended 12-core biopsy is not sufficient to exclude contralateral disease and cannot provide reliable, accurate prognostic information. Johnstone et al. [15] found that conventional prostate biopsy (6–12 cores) in 11 reported series comprising almost 800 patients with minimal unilateral disease on biopsy was unreliable compared to final pathology. Final pathologic assessment of prostatectomy specimens revealed a maximum tumor volume >10 cc in 3 series, extraprostatic extension in 10.5% of cases, a median positive surgical margin rate of 10.5%, subsequent discovery of Gleason grade 4 disease in 14% of patients, and bilateral disease in almost 80% of cases. Nogueira et al. [16] retrospectively analyzed data on 202 patients with low-risk criteria (no Gleason grade 4 or 5, a single involved core, <2 mm length, PSA density ≤0.10, and clinical stage ≤T2a) to evaluate the utility of an initial TRUS-guided biopsy and preoperative magnetic resonance imaging to predict indolent cancer (defined as pathologically organ-confined cancer ≤0.5 cc and without poorly differentiated elements). The final pathologic review revealed nonindolent cancer in 50% of specimens.

In recent years we and others have reported a trend towards an increasing number of dominant anterior prostatic tumors [17, 18]. A significant percentage of these cancers are located in the prostatic transition zone and are typically more difficult to detect by TRUS-guided biopsy or digital rectal examination and are poorly visualized on imaging [19, 20].

In a series of studies, investigators from our institution have carefully reexamined the histoanatomy of the anterior prostate, compared pathologic variables between transition zone and anterior peripheral zone tumors, and retrospectively determined the accuracy with which transition zone-directed needle biopsies detect clinically relevant transition zone tumors [7, 18, 21]. In a detailed histopathologic analysis of 197 anterior dominant tumors, emphasizing the variability in anterior prostatic anatomy from apex through base, to determine zone of origin and pathological staging, al-Ahmadie et al. showed that the majority of anterior dominant tumors in the prostate are actually of anterior peripheral zone origin [18].

These observations are pertinent in assessment of tumor location detected by needle biopsy. Given the difficulties in detecting anterior prostatic tumors on clinical examination, imaging studies, and needle biopsy, a number of investigators have examined the value of transition zone-directed needle biopsies in prostate cancer detection, with conflicting results. Surprisingly, however, few studies have correlated the cancer seen in these needle biopsies with that seen in prostatectomy specimens and/or the clinical relevance of these tumors [22, 23]. Characterization of cancers using template-based prostate biopsy strategies depends largely on the density of sampling. New biopsy techniques that map the prostate systematically can limit errors and provide information regarding stage, grade, tumor volume, and spatial distribution of cancer inside the prostate, potentially improving the outcomes of conservative management options including active surveillance and focal therapy. Barzell and Melamed [3] compared results of three-dimensional TMB after TRUS-guided biopsy to further detect clinically significant tumors before initiating focal therapy. They reported on 80 patients who underwent TMB with a median of 69 cores taken per patient (1.88 cores per mL of prostate). Of these, 43 patients (54%) with a diagnosis of unilateral disease on TRUS-guided biopsy actually had bilateral disease and were therefore unsuitable for unilateral focal therapy. Similar conclusions were obtained by Onik et al. [4] in a study of 180 patients with unilateral cancer on TRUS-guided biopsy who were considering conservative management. After restaging with three-dimensional TMB, 110 (61.1%) were positive bilaterally and 41 (22.7%) had Gleason score ≥7.

TMB has been shown to have better accuracy in the anterior prostate than traditional TRUS-guided prostate biopsies. In a study of 118 men, Furuno et al. [24] compared standard transrectal sextant biopsy with an extended transperineal ultrasound-guided template prostate biopsy, obtaining an average of 18 cores. Their results suggest that transrectal sextant biopsies missed more tumors in the anterior than in the posterior region of the gland. By contrast, the transperineal template-guided mapping technique detected cancer equally well in the anterior and posterior regions. A more recent study by Ayres et al. [25] supported this finding that standard TRUS biopsy often undersamples the anterior portion of the gland. In 101 men on active surveillance with low-risk prostate cancer, 34% of men were found to have higher-risk prostate cancer on subsequent TMB, with about half of these men having disease in the anterior portion of the gland.

Recently Taira et al. [5] showed, in a series of 373 consecutive men, that TMB had a high detection rate as initial biopsy (75.9%) and as a repeat biopsy (46.9%). Over half of all cancers found were Gleason ≥7. Cancer was identified in all of the regions sampled in the group that underwent TMB as their initial biopsy. In patients with multiple prior TRUS-guided biopsies, cancer was most commonly found in the anterior and apical aspects of the prostate, demonstrating the ability of TMB to diagnose tumors in those clinically significant locations.

Numao et al. [26] compared the accuracy of three-dimensional 26-core (3D26) prostate biopsy with extended transrectal 12-core and transperineal 14-core biopsy in predicting Gleason pattern 4 or 5 in the final RP specimens of 143 consecutively treated men. They demonstrated that the 3D26 biopsy accurately predicted the presence of Gleason pattern 4 or 5 cancers in prostatectomy specimens with a higher concordance rate (92.3%) than that achieved with extended transrectal 12-core biopsy or transperineal 14-core biopsy (83.5% and 85%, resp.).

In this very select group of 17 patients—which only included patients who had findings after TMB that warranted subsequent RP—TMB in this setting demonstrated reasonable diagnostic accuracy for detecting tumors in the final specimen, with a positive predictive value of 0.90. This approaches the performance estimated from computer modeling studies of TMB [27]. From the total 68 quadrants, there were only four false negative quadrants (negative on TMB but tumor was identified in the final pathologic specimen). Of these, only two quadrants represented significant tumors, both of which were located in the anterior quadrant.

While the natural history of different prostate cancer foci remains unknown, evidence suggests that an index tumor may determine the biological potential for malignancy. By convention, such tumors have been defined as those with volume >0.5 cc and/or Gleason ≥7 signifying clinically significant disease [28–30]. The positive and negative predictive values of TMB for such tumors in this series (0.66 and 0.86, resp.) are encouraging, although not as accurate as would be hoped. This may not be unexpected, however, given the spatial resolution limitations with biopsy to identify lesions defined, in part, not by geometry but grade.

TMB may help identify men who are considering active surveillance but who have significant cancers typically missed on traditional TRUS-guided prostate biopsies, thereby allowing the clinician to risk-stratify patients with increasing confidence. Likewise, focal therapy may become a more attractive treatment option if the treating physician can more confidently identify the index lesion, confirming that a significant contralateral lesion is not being ignored. Despite being reasonably accurate in detecting tumors, there remain certain limitations of TMB, evidenced by the several false positives and false negatives seen in our series, which may indicate localization inaccuracies with needle placement despite the use of template and ultrasound guidance. Discrepancies between TMB and final pathology specimen could be due to the variation in spatial orientation between the anatomical position of the prostate in vivo compared to the prostate specimen ex vivo. In some cases, the biopsy needle can change the orientation of the prostate, thus sampling contiguous areas. Hemorrhage during the procedure, deformation of the gland by the numerous needles passing through it, and needle tip excursion may also explain the discrepancies. Finally, latest developments in multiparametric magnetic resonance imaging (mpMRI) techniques for prostate imaging and targeted biopsy techniques utilizing ultrasound fusion approach seem promising in improving our ability to not only accurately identify lesions within the prostate, but also target it during transrectal ultrasound-guided biopsy. Although the accuracy is still lacking in low-risk lesions, which would be the main focus for the employment of such technology in this population of patients, the convergence of all these techniques will likely overcome these limitations and allow for the establishment of safe organ-sparing management strategies in the near future.

The primary limitations of this study are the small size and the select nature of the cohort. Inclusion was based primarily on all patients who proceeded to prostatectomy after undergoing TMB, which represented a minority of all men who have had TMB at our institution (17 out of 78). We do not know if these results are applicable to patients that continued on active surveillance based on the TMB result. However, without whole-mount pathological examination of these men's prostates, it is impossible to conduct such an analysis. This series is also limited as an exploratory study which focused exclusively on the performance characteristics of TMB in the detection and characterization of prostate cancers and not on the clinical outcomes or correlates that fall outside of this intent. To the best of our knowledge, however, no similar studies have been reported in the literature comparing in this way the accuracy of preoperatively performed TMB to subsequent radical prostatectomy specimens from the same patients. This study evaluates a unique cohort of patients providing data of special interest to researchers in this field.

## 5. Conclusion

The advocated use of a TMB template for identifying, characterizing, and spatially localizing clinically significant prostate tumors is supported by these results that demonstrated a sensitivity of over 90% in the detection of such tumors. Justification for this highly more invasive procedure on clinical grounds, however, remains to be prospectively explored. The application of TMB for pretreatment planning, particularly for organ-sparing approaches to prostate cancer management, deserves consideration.

## Figures and Tables

**Table 1:** Patient characteristics at initial TRUS-guided biopsy (*N* = 17).

PSA (ng/mL), median (IQR)	5.6 (4.1–7.0)
Clinical stage, *n* (%)	
T1c	13 (76)
T2a	2 (12)
No cancer	2 (12)
Number of cores taken at TRUS biopsy, *n* (%)	
6	1 (6)
10–12	10 (59)
>12	6 (35)
Number of positive cores, *n* (%)	
0	2 (12)
1	13 (76)
2	2 (12)
Length of cancer in positive cores (mm), median (IQR)	1.5 (0.7–2.0)
% of positive cores, *n* (%)	
0%	2 (12)
1–5%	11 (65)
6–10%	3 (18)
>10%	1 (6)
Biopsy Gleason score, *n* (%)	
2 + 3	1 (6)
6	11 (65)
7 (3 + 4)	3 (18)
No cancer	2 (12)
Laterality	
Right	8 (47)
Left	6 (35)
Bilateral	1 (6)
No cancer	2 (12)
Time between TRUS biopsy and prostatectomy (months), median (IQR)	4 (3–6)

**Table 2:** Overall characteristics of TMB and prostatectomy specimens (*N* = 17).

*Transperineal Mapping Biopsy (TMB) *	
Number of cores taken, *n* (%)	
17–75	9 (53)
76–114	8 (47)
Number of positive cores, *n* (%)	
0	0 (0)
1–5	5 (29)
6–10	9 (53)
>10	3 (18)
Core density (number of cores/specimen volume), median (IQR)	1.26 (1.08–1.51)
Length of cancer in positive cores (mm), median (IQR)	12.8 (9.5–16.1)
Gleason score, *n* (%)	
6	5 (29)
7 (3 + 4)	10 (59)
7 (4 + 3)	1 (6)
9 (4 + 5)	1 (6)
Laterality, *n* (%)	
Right	2 (12)
Left	2 (12)
Bilateral	13 (76)
*Prostatectomy Specimen *	
Volume (cc), median (IQR)	47 (37–65)
Gleason score, *n* (%)	
6	3 (18)
7 (3 + 4)	10 (59)
7 (4 + 3)	3 (18)
8	1 (6)
Laterality	
Right	2 (12)
Left	2 (12)
Bilateral	13 (76)
Maximum area of cancer (mm^2^), median (IQR)	0.52 (0.22–0.92)
Maximum diameter of cancer (mm), median (IQR)	1.21 (0.91–1.49)

**Table 3:** Characteristics of TMB and prostatectomy specimen for the four prostate quadrants.

	Quadrant*			
	Left Anterior *N* = 17	Right Anterior *N* = 17	Left Posterior *N* = 17	Right Posterior *N* = 17
*Transperineal Mapping Biopsy (TMB) *				
Number of cores taken, *n* (%)				
2–18	9 (53)	10 (59)	10 (59)	10 (59)
20–33	8 (47)	7 (41)	7 (41)	7 (41)
Number of positive cores, *n* (%)				
0	6 (35)	5 (29)	8 (47)	5 (29)
1	3 (18)	4 (24)	1 (6)	2 (12)
2	2 (12)	4 (24)	3 (18)	2 (12)
3	0 (0)	0 (0)	2 (12)	6 (35)
>3	6 (35)	4 (24)	3 (18)	2 (12)
*Length of cancer in positive cores (mm), median (IQR)	3.2 (2.0–17.8)	4.8 (1.3–7.7)	3.0 (2.0–6.3)	6.0 (4.4–7.2)
Gleason score, *n* (%)				
Negative	6 (35)	5 (29)	8 (47)	5 (29)
6	7 (41)	7 (41)	5 (29)	5 (29)
7 (3 + 4)	4 (24)	5 (29)	4 (24)	5 (29)
7 (4 + 4)	0 (0)	0 (0)	0 (0)	1 (6)
9 (4 + 5)	0 (0)	0 (0)	0 (0)	1 (6)
*Prostatectomy Specimen *				
Gleason score				
Negative	6 (35)	6 (35)	7 (41)	7 (41)
6	3 (18)	5 (29)	5 (29)	4 (24)
7 (3 + 4)	7 (41)	6 (35)	3 (18)	3 (18)
7 (4 + 3)	1 (6)	0 (0)	2 (12)	2 (12)
8	0 (0)	0 (0)	0 (0)	1 (6)
**Area of cancer (mm^2^), median (IQR)	0.42 (0.19–1.14)	0.45 (0.11–0.87)	0.24 (0.13–0.41)	0.24 (0.18–0.48)
**Diameter of cancer (mm), median (IQR)	1.15 (0.79–1.66)	1.20 (0.58–1.49)	0.87 (0.51–0.97)	0.84 (0.73–1.08)

*A total of 68 quadrants from 17 patients were examined. **Among the 42 quadrants with cancer present.

**Table 4:** Sensitivity and specificity of TMB for detection of prostate cancer, overall and separately by quadrant, using RP specimen pathology as gold standard.

			Cancer in specimen		Sensitivity % (95% CI)	Specificity % (95% CI)	Positive predictive value (95% CI)	Negative predictive value (95% CI)
			Yes	No				
Overall								
	Cancer in biopsy	Yes	38	4	86 (72–94)	83 (62–95)	90 (77–97)	77 (56–91)
		No	6	20				

By Quadrant								
Left anterior	Cancer in biopsy	Yes	10	1	91 (58–99)	83 (35–99)	91 (58–99)	83 (35–99)
		No	1	5				
Right anterior	Cancer in biopsy	Yes	9	2	75 (42–94)	60 (14–94)	81 (48–97)	50 (11–88)
		No	3	3				
Left posterior	Cancer in biopsy	Yes	9	1	100 (66–100)	88 (47–99)	90 (55–99)	100 (59–100)
		No	0	7				
Right posterior	Cancer in biopsy	Yes	10	0	83 (51–97)	100 (47–100)	100 (69–100)	71 (29–96)
		No	2	5				

By Anterior/posterior								
Left Anterior + Right Anterior	Cancer in biopsy	Yes	19	3	83 (61–95)	73 (39–93)	86 (65–97)	67 (34–90)
		No	4	8				
Left Posterior + Right Posterior	Cancer in biopsy	Yes	19	1	90 (69–98)	92 (63–99)	95 (75–99)	85 (57–98)
		No	2	12				

By Right/left								
Right Anterior + Right Posterior	Cancer in biopsy	Yes	19	2	79 (57–92)	80 (44–97)	90 (69–98)	62 (31–86)
		No	5	8				
Left Anterior + Left Posterior	Cancer in biopsy	Yes	19	2	95 (75–99)	86 (57–98)	90 (69–98)	92 (63–99)
		No	1	12				
